# Unraveling Burnout, Diabetes Distress, and Depression: Insights into Their Impact on Adults with Diabetes

**DOI:** 10.1192/j.eurpsy.2025.482

**Published:** 2025-08-26

**Authors:** S. Er, M. Murat Mehmed Ali, G. Anataca, S. Çelik

**Affiliations:** 1 Department of Mental Health and Psychiatric Nursing, Istanbul University – Cerrahpaşa, Florence Nightingale Faculty of Nursing; 2 Psychiatric Nursing Department, University of Health Sciences, Hamidiye Faculty of Nursing; 3 Kanuni Sultan Suleyman Education and Research Hospital, Istanbul Health Sciences University; 4 Department of Internal Medicine Nursing, University of Health Sciences, Hamidiye Faculty of Nursing, Istanbul, Türkiye

## Abstract

**Introduction:**

Diabetes burnout refers to the physical and emotional fatigue resulting from the challenges of living with and managing diabetes. It is a prevalent issue that significantly affects diabetes care. Unfortunately, it often receives less attention and is frequently underestimated.

**Objectives:**

This study examined the prevalence and predictors of burnout, diabetes distress and depression in adults with diabetes mellitus.

**Methods:**

In this cross-sectional study, investigated the Turkish validity and reliability of the Diabetes Burnout Scale and determined prevalence and predictors burnout, diabetes stress, and depression among adults with DM. The study sample consisted of 315 adults and a Personal Information Form, the Diabetes Burnout Scale, the Diabetes Distress Scale, and the Patient Health Questionnaire-8 were administered. Data analysis involved descriptive tests, Pearson correlation analysis, and the use of SPSS (v.29).

**Results:**

As a result of confirmatory factor analysis, it was determined that the Turkish version of the Diabetes Burnout Scale had 3 sub-dimensions and consisted of 12 items. The Cronbach alpha reliability coefficient of the total scale was found to be 0.814. Participants had a mean (±SD) age of 48.63 (±8.23) years, with a majority (54.6%) being male and (±93.7%) diagnosed with type 2 diabetes mellitus. The median duration of diabetes among them was 4.41 (±2.48) years, and their median HbA1c level was 8.99 (±0.68). The prevalence rates for burnout, diabetes distress, and depression were found as 2.69 (±0.28), 4.64 (±0.40), and 15.20 (±3.88), respectively. Additionally, the total burnout score showed a positive correlation with both the diabetes distress score (r = 0.556, p = 0.033) and depression (r = 0.325, p = 0.027).

**Conclusions:**

The study revealed a high prevalence of burnout, distress, and depression, highlighting the need for a prevention strategy. Monitoring high-risk groups for pre-diabetes and diabetes is crucial for informing health programs and resource distribution to manage the condition effectively.

**Disclosure of Interest:**

None Declared

